# Assessing the Incidence of New-onset Diabetes Mellitus with Statin Use: A Systematic Review of the Systematic Reviews and Meta-analyses

**DOI:** 10.17925/EE.2022.18.2.96

**Published:** 2022-11-29

**Authors:** Harmanjit Singh, Pallavi Sikarwar, Supreet Khurana, Jatin Sharma

**Affiliations:** * These authors have contributed equally to this work and share first authorship.; 1. Department of Pharmacology, Government Medical College and Hospital, Chandigarh, India; 2. MBBS Student, Government Medical College and Hospital, Chandigarh, India; 3. Department of Neonatology, Government Medical College and Hospital, Chandigarh, India; 4. Department of Pharmacology, All India Institute of Medical Sciences, New Delhi, India

**Keywords:** Atorvastatin, cardiovascular diseases, diabetes mellitus, HMG-CoA reductase inhibitors, meta-analysis

## Abstract

Statin use has been linked with new-onset diabetes mellitus (NODM). In the present systematic review, we aimed to determine the incidence of NODM with statin use by assessing and summarizing the data generated by different systematic reviews and metaanalyses published on this topic. We conducted a systematic review of systematic reviews and meta-analyses using a pre-defined study protocol. Two authors independently performed a literature search using PubMed, Embase and the Cochrane Central Register of Controlled Trials (CENTRAL) for studies reporting data on statin use and NODM incidence and screened and extracted data for the outcomes of interest. The Assessing the Methodological Auality of Systematic Reviews 2 (AMSTAR 2) checklist was used to evaluate the quality of the included systematic reviews and meta-analyses. The initial search yielded 621 potential records, and 16 relevant systematic reviews and meta-analyses were included in the present systematic review. The included studies showed an increase in the risk of NODM with statin use. In particular, rosuvastatin and atorvastatin were associated with NODM in many systematic reviews or meta-analyses; however, pravastatin and pitavastatin were found to be associated with lower or no risk. We observed a positive trend of development of NODM with statin use became more evident with advancing years as more number of studies were added. Intensive doses of statins and use in older subjects were found to be important risk factors for NODM. Finally, the quality assessment revealed that the included systematic reviews and metaanalyses were of critically low or low quality. We concluded that statin use carries a risk of causing NODM. Statins should not be discouraged in anticipation of NODM. However, glycaemic monitoring should be encouraged with the on-going statin therapy. Furthermore, clinical studies addressing the use of statins and the incidence of NODM as their primary objective should be planned.

Recently, concern has been raised that statin therapy may be associated with new-onset diabetes mellitus (NODM), especially given the wide usage of statins in the treatment of dyslipidaemia and cardiovascular diseases (CVD). Statins have been found to affect not only the functioning of the beta cells of the pancreas, but their use also leads to insulin resistance.^1^ Beta cell function may be affected by statins through various pathways, including:

- blockade of insulin release mediated by glucose transporter 2 (GLUT2)
- decreased insulin secretion due to energy depletion caused by a decreased production of ubiquinone
- decreased glucose transporter 4 (GLUT4) in adipocytes
- inhibition of the 3-hydroxy-3-methylglutaryl coenzyme A (HMG-CoA) reductase, which leads to increased uptake of plasma low-density lipoprotein cholesterol (LDL-C) that enters and inhibits glucose-mediated insulin release in cells.^1,2^

On the other hand, resistance to insulin in cells has been recently attributed to the activation of the NOD-like receptor family pyrin domain containing 3 (NLRP3) inflammasome, which in turn activates interleukin 1β-causing insulin resistance.^1,3^

The exploration of the diabetogenic effect of statin therapy is on-going worldwide, and the results of such investigations may have a major impact on the management of dyslipidaemias and CVD with statins. An observational study analysed the data collected by IMS Health between February 2006 to January 2010 and registered an increase of more than 2.3 times in the fraction of the Indian population receiving statins daily, which rose from 3.35% to 7.78% during this period.^4^

A landmark study by Culver et al. included 153,840 women without diabetes mellitus (DM) and found that statin use at baseline is associated with an increased risk of NODM (hazard ratio (HR) 1.71, 95% confidence interval [CI] 1.61–1.83).^5^ This association was observed even after adjusting for covariates and was found to be a class effect of the statins. This study was important, as it raised many questions on the long-term use of statins and the risk of statin-caused DM. Considering that statins are widely used for the treatment of dyslipidaemia and CVD, the number of patients using statins is set to increase following the concomitant rise in the CVD states.^6^ In this context, the association of statin therapy with NODM in patients previously free from DM becomes important.^7–9^ A few other studies have shown that the diabetogenic effect of statin is dose dependent, with the risk of NODM increasing at higher doses.^10,11^ In addition to the dose, the diabetogenic effect of statins can also be determined by the duration of statin use. A population-based control study has shown that the risk of NODM is higher in new statin users.^9^ Moreover, a study by Dormuth et al. concluded that there is a significant risk of NODM in the first 2 years of therapy, with higher potency statins and maximum risk seen in the first 4 months of therapy.^12^

From the preliminary reports mentioned above, it can be concluded that statin use is linked with NODM; however, different studies obtained varying results. As systematic reviews and meta-analyses are considered the best way to generate evidence on any topic, we performed a systematic review of the systematic reviews and metaanalyses published on statin use and NODM incidence. In the present systematic review, we aimed to determine the incidence of NODM with statin use by assessing and summarizing the data generated by different systematic reviews and meta-analyses. Compared with the conventional research studies, the information generated by gathering the data from systematic reviews and meta-analyses can help to understand better the relationship between statin use and the risk of NODM.

## Methods

We conducted a systematic review of systematic reviews and metaanalyses using a pre-defined study protocol, which we registered on Prospero (Prospero registration number CRD42021260658). The study was prepared and reported according to the Preferred Reporting Items for Systematic Reviews and Meta-Analyses (PRISMA) statement for systematic reviews and meta-analyses.^13^

### Search strategy and selection criteria

Two authors (HS and PS) independently performed a comprehensive literature search for relevant systematic reviews and meta-analyses of studies reporting the data on statin use and incidence of NODM using PubMed, Embase and the Cochrane Central Register of Controlled Trials (CENTRAL). We included systematic reviews and meta-analyses published in English from study inception to November 2019. We used keywords such as ‘statins’, ‘HMG-CoA reductase inhibitor’, ‘new-onset diabetes mellitus’, ‘dyslipidemia’ and ‘cardiovascular disease’. These keywords were combined using the filters AND/OR wherever applicable. We also searched the bibliography of the selected articles to retrieve other potential studies.

### Study selection

The same two authors (HS and PS) independently assessed the title, abstracts, keywords and full text of the potential studies for inclusion in the search results. Systematic reviews and meta-analyses addressing our research question were included in this systematic review. We did not include articles such as case reports, randomized or non-randomized clinical trials, observational studies, review articles, hypotheses and conference abstracts/presentations. Following are the parts of our research question, and the keywords and literature search were planned accordingly:

- Participants (P): Statin users who are included in different studies in the respective systematic reviews and meta-analyses;
- Intervention (I)/Exposure: Any of the statin drugs used;
- Comparator (C): Participants in the control groups included in different studies of the respective systematic reviews and meta-analyses receiving either placebo or any other active control;
- Outcome/s (O): Assessing the incidence of NODM with statin use and looking for the information with respect to the following: type of statin and incidence of NODM, dose of statin and its relationship with NODM, and duration of statin use and its relation with NODM.

### Data extraction (selection and screening)

Microsoft Excel was used to develop a data extraction spreadsheet after piloting and adjusting it by applying it to a small selection of studies. Two authors (HS and PS) independently screened and extracted data for outcomes of interest and performed methodological and quality assessments. Any disagreements were discussed between the two authors and were resolved with the help of two other authors (SK and JS). The data were summarized in tabulated form, and details of the included systematic reviews and meta-analyses were presented with respect to authors and year; types of studies included in the systematic reviews/ meta-analyses; total number of participants included in the systematic reviews/meta-analyses; methodology and results of the systematic reviews/meta-analyses including the quantitative data synthesis, risk of bias, publication bias and funding information.

### Quality of the included systematic reviews and meta-analyses

We used the Assessing the Methodological Quality of Systematic Reviews 2 (AMSTAR 2) checklist to evaluate the quality of the included systematic reviews and meta-analyses.^14^ The risk of bias assessment was not performed since we included systematic reviews and meta-analyses in our systematic review and not randomized controlled trials (RCTs). Data from the systematic reviews and meta-analyses were summarized in a tabulated form, and we did not perform a quantitative data synthesis as our work was based on the findings of other published systematic reviews and meta-analyses. Similarly, no specific statistical tests or techniques were required for this systematic review.

## Results

**Figure 1:**
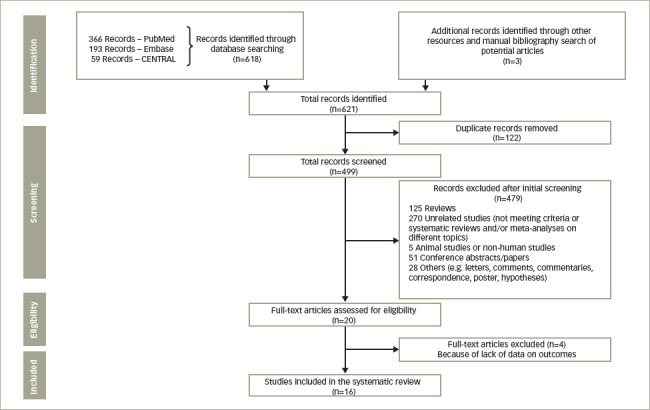
PRISMA flow diagram

The initial search yielded 621 potential records. After applying the inclusion criteria, removing duplicates and screening the potential records, we selected 16 relevant systematic reviews and meta-analyses for this systematic review.^15–30^ The stepwise inclusion/exclusion of the articles/records has been given in the PRISMA flow diagram (*Figure 1*).

The included systematic reviews contained the information of many thousands of patients receiving either the statin medication or the control group intervention. The exact number of participants considered in our systematic review could not be provided since most of the included systematic reviews and meta-analyses comprised some of the same research studies depending on their inclusion criteria and the year they were conducted and published.

*CENTRAL = Cochrane Central Register of Controlled Trials.*

Most of the systematic reviews and meta-analyses included RCTs to gather information on statin use and incidence of NODM; however, few of them included observational studies as well. A summary of the included systematic reviews and meta-analyses with respect to authors and year, types of studies included, total number of participants, their methodology and results including those of the quantitative data synthesis, risk of bias, publication bias and funding information, and their quality is given in *Table 1* (*supplementary material*).^15–30^

### Findings of the included systematic reviews and meta-analyses

The meta-analysis of RCTs performed by Coleman et al. (n=39,791, follow-up range 2.7–6.0 years) concluded that statins did not significantly alter the development of DM (risk ratio [RR] 1.03, 95% CI 0.89–1.19).^15^ The subgroup and sensitivity analyses did not significantly change the results.

In a meta-analysis of hypothesis-testing RCTs (n=57,593) performed by Rajpathak et al., a small increase in DM risk (RR 1.13, 95% CI 1.03–1.24; p=0.007) was observed with no evidence of heterogeneity.^16^ However, this estimate was no longer significant once a hypothesis-generating trial was included (RR 1.06, 95% CI 0.93–1.25; p=0.38), and it also showed significant heterogeneity.

In the meta-analysis of RCTs performed by Sattar et al., 2,226 patients taking statins and 2,052 taking the control treatment developed DM during a mean of 4 years.^17^ Statin therapy led to a 9% increased risk for incident DM (odds ratio [OR] 1.09, 95% CI 1.02–1.17), and a metaregression showed that the risk of developing DM with statins was highest in older participants.

The meta-analysis performed by Preiss et al. included 5 statin RCTs with 32,752 participants without DM at baseline, 2,749 of which developed NODM (intensive-dose group: n=1,449; moderate-dose group: n=1,300) with a mean follow-up of 4.9 years (OR 1.12, 95% CI 1.04–1.22; I^2^=0%).^18^

In the meta-analysis performed by Cai et al., 14 RCTs comprising 95,102 non-diabetic participant were included.^19^ The NODM risks were found to be increased by 33.0% (OR 1.33, 95% CI 1.14–1.56; I^2^=7.7%) and 16.0% (OR 1.16, 95% CI 1.06–1.28; I^2^=0.0%) when the intensified target LDL-C levels were ≤1.8 mmol/L and 1.8–2.59 mmol/L, respectively. The risk of NODM did not increase when the target LDL-C level was ≥2.59 mmol/L (OR 1.01, 95% CI 0.92–1.10; I^2^ = 0.0%).

In the meta-analysis conducted by Navarese et al., 17 RCTs with 113,394 patients were included.^20^ Pravastatin 40 mg/day was found to be associated with the lowest risk of developing DM compared with placebo (OR 1.07, 95% credible interval 0.86–1.30), while rosuvastatin 20 mg/day was found to be associated with a 25% increased risk compared with placebo (OR 1.25, 95% credible interval 0.82–1.90), and atorvastatin 80 mg/day showed an intermediate impact (OR 1.15, 95% credible interval 0.90–1.50). The authors concluded that different statins and their doses have different potentials to cause NODM, and higher doses are linked to a higher risk.

In the meta-analysis by Naci et al., which comprised 135 trials and 246,955 participants with an average follow-up of 1.3 years, rosuvastatin was found to lead to significantly higher odds of DM compared with placebo (OR 1.16, 95% CI 1.02–1.31; I^2^=0.0%).^21^ However, the drug-level network meta-analysis did not achieve statistical significance. The authors also observed that there was no significant difference between individual statins in terms of DM incidence, but rather it seems to be a class effect (OR 1.09; 95% credible intervals, 1.02–1.16; I^2^=2.8%).

In the meta-analysis performed by Finegold et al., 14 primary prevention RCTs with 46,262 subjects and 15 secondary prevention RCTs with 37,618 subjects were included in the final analysis.^22^ Among the 14 primary prevention trials, statin therapy increased the absolute risk of developing DM by 0.5% compared with placebo (95% CI 0.1–1.0%; p=0.012). Therefore, the authors observed that the development of DM was significantly higher on statins than on placebo (1 in 5 of new cases were actually caused by statins) and that higher doses produced a more detectable effect. Conversely, only one of the 15 secondary prevention trials reported the rates of DM development, but this effect was not significant (95% CI -0.5 to 1.6%; p=0.387).

In the meta-analysis conducted by Swerdlow et al., in 129,170 individuals free from type 2 diabetes mellitus (T2DM) from 20 RCTs, statins were found to increase the odds of NODM (all trials: OR 1.12, 95% CI 1.06–1.18; placebo or standard care-controlled trials: OR 1.11, 95% CI 1.03–1.20; intensive-dose versus moderate dose trials: OR 1.12, 95% CI 1.04–1.22).^23^ No significant heterogeneity was observed.

The meta-analysis performed by Teng et al. included 8 trials (n=25,952) and found that statins significantly reduced the risks of composite major adverse cardiovascular events, nonfatal myocardial infarction and total myocardial infarction; however, no significant differences were observed in myalgia, elevation of hepatic transaminases, NODM (RR 1.07, 95 % CI 0.77–1.48), serious adverse events and discontinuation due to adverse events.^24^

The meta-analysis performed by Vallejo-Vaz et al. included 15 RCTs assessing the effects of pitavastatin on glycaemia and NODM and involving 4,815 patients free from DM (3,236 allocated to pitavastatin and 1,579 to control).^25^ No significant differences associated with pitavastatin were observed for fasting blood glucose, haemoglobin A1c (HbA1c) and NODM (RR 0.70, 95% CI 0.30–1.61; I^2^=0.00%) compared with placebo. No significant differences were found whether the authors considered a short-term or a longer follow-up.

In the meta-analysis performed by Thakker et al., 29 RCTs were included. (n=163,039; patients free from DM: n=141,863).^26^ They found that statins significantly increased the likelihood of developing NODM by 12% (pooled OR 1.12, 95% CI 1.05–1.21; I^2^=36%; p=0.002; 18 RCTs). In the network meta-analysis, atorvastatin 80 mg was found to be associated with the highest risk of developing DM (OR 1.34, 95% CI 1.14–1.57), followed by rosuvastatin (OR 1.17, 95% CI 1.02–1.35).

In their meta-analysis comprising 14 studies with a total of 94,943 participants, Rahal et al. found that 2,392 subjects developed NODM in the statin group and 2,167 in the placebo group during a 4-year follow-up.^27^ The OR of NODM incidence with overall statin therapy was significantly higher compared with placebo (OR 1.11, 95% CI 1.00–1.20; p=0.007). The subgroup analysis revealed that atorvastatin (OR 1.29, 95% CI 1.0–1.6; p=0.042) and rosuvastatin (OR 1.17, 95% CI 1.0–1.3; p=0.01) were significantly associated with the risk of NODM.

In the meta-analysis of observational studies performed by Casula et al., 20 studies (18 cohort and 2 case-control studies) were included.^28^ NODM risk was found to be higher in statin users than non-users (relative risk 1.44, 95% CI 1.31–1.58). High heterogeneity between studies was observed (I^2^=97%). A class effect, from rosuvastatin (relative risk 1.61, 95% CI 1.30–1.98) to simvastatin (relative risk 1.38, 95% CI 1.19–1.61), was observed regarding the risk of NODM.

In the meta-analysis performed by Wang et al., 14 RCTs (participants free from DM: n=95,102) were included.^29^ From these studies, 8 trials meeting the target LDL-C levels of ≤100 mg/dL or an LDL-C reduction of at least 30% were extracted. The authors found an overall risk of incident diabetes increased by 11% (OR 1.11, 95% CI 1.03–1.20). The intensive LDL-C-lowering statin group showed an 18% increase in the likelihood of developing DM compared with placebo (OR 1.18, 95% CI 1.10–1.28; I^2^=0.0%). The authors also observed that, over 4 years of statin therapy, there was one additional case of DM per 137 users with a 30–40% relative reduction in LDL-C and one per 108 statin users with a 40–50% relative reduction in LDL-C.

In the meta-analysis performed by Kamran et al., 11 studies were included (N=236,864; statin group: n=56,053; control group: n=180,811).^30^ In the statin group, 4,732 subjects developed DM compared with 10,447 subjects in the control group (fixed effects model: pooled OR 1.61, 95% CI 1.55–1.68; random effects model: pooled OR: 1.92, 95% CI 1.64– 2.25; p<0.001). These results suggest a significant positive association between statin use and NODM. High heterogeneity was observed for the studies included (Q statistic=103.5; p<0.001).

### Quality of the included systematic reviews and meta-analyses

Most of the systematic reviews and meta-analyses included in this systematic review were found to be of critically low to low quality as evaluated by the AMSTAR 2 checklist (*Table 1*, *supplementary material*).

## Discussion

NODM is an emerging issue associated with the use of statins. Statins are one of the most important agents for the treatment of dyslipidaemia and for the primary and secondary prevention of CVD.

In our systematic review, we focused on published systematic reviews and meta-analyses to collect and collate information regarding the use of statins and the risk of NODM since systematic reviews and metaanalyses are based on multiple research studies and can provide valuable information on this association.

With the exception of some of the systematic reviews and meta-analyses included in this systematic review,^15,24,25^ the majority of the reports found that there is an increase in the risk of NODM with the use of statins; however, the evidence is still emerging. Most of the systematic reviews and meta-analyses included in this systematic review were based on RCTs or observational studies where the primary objective was not to investigate the association between statin use and the incidence of NODM. The meta-analysis by Coleman et al. is fairly old (2008), and it included the initial studies that were published on the topic in their analysis.^15^ However, recent reports are showing a positive association between statins and the risk of NODM. In the meta-analysis by Vallejo-Vaz et al., the data only focused on pitavastatin, and other statins that are implicated in increasing the risk of NODM, such as rosuvastatin and atorvastatin, were excluded.^25^ However, the majority of the included systematic reviews and meta-analyses have raised this concern and demanded the monitoring of the patients receiving statins with respect to glycaemic parameters.

The systematic reviews and meta-analyses in this systematic review have pointed out certain important risk factors that are more likely to be linked with the development of DM. These reports have shown that higher or intensive doses of statins are more likely to be associated with the risk of NODM; hence, subjects receiving higher or intensive doses should be closely monitored. The reports also identified risk factors, such as older subjects and intensity of LDL-C reduction, that are more commonly associated with the development of statin-induced NODM.^18,19,29^ Based on the reports included in our systematic review, it appears that NODM is a class effect of the statin group; however, the meta-analyses conducted by Navarese et al. reported that pravastatin is not associated with NODM compared with placebo (OR 1.07, 95% CI 0.86–1.30).^20^

The findings described above regarding the differential diabetogenic effect of individual statins was confirmed by a network meta-analysis recently performed by Seo et al.^31^ The authors compared the risk of NODM between subjects who received pitavastatin and atorvastatin or rosuvastatin by analysing the data of electronic health records collected from 10 hospitals (n=14,605,368 patients). In the sub-analysis performed by the authors, pitavastatin was found to be associated with a lower risk of NODM compared with atorvastatin and rosuvastatin (HR 0.72, 95% CI 0.59–0.87).

Furthermore, statin-induced NODM seems to be dose dependent as most of the reports have shown that higher doses of statins such as atorvastatin and rosuvastatin are more commonly associated with the risk of developing DM. Another risk factor of statin-induced NODM is the target LDL-C reduction or the intensity of LDL-C lowering. Reports included in our systematic review have shown that more aggressive LDL lowering led to a higher risk of developing DM.^19,29^ Hence, in both situations cited above, it is imperative to monitor the glycaemic parameters so that the risk can be assessed and further modifications can be made to the therapy if needed.

This association between statin use and disturbed glycaemic control could be more detrimental in individuals who already have T2DM, as many patients with T2DM also suffer from CVD or dyslipidaemia.^32^ A network meta-analysis conducted by Cui et al. investigated the impact of statin therapy on glycaemic control in patients with T2DM.^32^ The authors found that statin therapy led to an increase in HbA1c compared with placebo. They also found that moderate-intensity pitavastatin improved glycaemic control and that high-intensity atorvastatin worsened it. In this case, the clinicians should assess the risk of NODM by carefully monitoring these subjects’ blood glucose and HbA1c and implement timely measures to address this risk.

In a large retrospective study recently performed in the USA, Ziganshina et al. investigated whether baseline HbA1c level is a significant and independent risk factor for increasing the risk of NODM.^33^ They concluded that an HbA1c of between 6.0% and 6.4% is an independent risk factor for the development of DM, and, in this view, other risks from statin therapy may no longer be significant. However, this was a retrospective study, so further research, by carefully selecting the cohorts based on baseline HbA1c levels and evaluating the impact of statin therapy in developing NODM, is needed.

We could not find any conclusive evidence associating the duration of statin use and the risk of NODM. However, the findings of our systematic review are in agreement with a cohort study conducted by Ko et al., which found that, in a 3.9-year follow-up, subjects who had ever used statins had a significantly higher risk of developing DM compared with those who had never used statins (13.4 versus 6.9 events per 1,000 person-years; adjusted HR 1.88, 95% CI 1.85–1.93).^34^ It was also found that, with an increase in the duration of statin use, the corresponding risk of DM was proportionally increased (<1 year: adjusted HR 1.25, 95% CI 1.21–1.28; 1–2 years: adjusted HR 2.22, 95% CI 2.16–2.29; >2 years: adjusted HR 2.62, 95% CI 2.56–2.67), and the risk of DM was also associated with a higher intensity and cumulative statin dosing (higher intensity (adjusted HR 1.75, 95% CI 1.71–1.78 for low-to-moderate potency and HR 2.31, 95% CI 2.26–2.37 for high potency) cumulative dosing of statin (HR 1.06, 95% CI 1.02–1.10 for low-tertile, HR 1.74, 95% CI 1.70–1.79 for middle-tertile, and HR 2.52, 95% CI 2.47–2.57 for high-tertile of defined-daily-disease).

We checked the quality of the included studies with the AMSTAR 2 checklist, a very stringent instrument based on the latest recommendations with respect to systematic reviews and meta-analyses.^35,36^ Most of the reports included in our systematic review were performed even before the inclusion of those latest recommendations and were also based on older studies. This is probably the reason for the critically low or low quality of the included systematic reviews and meta-analyses. This scenario may improve with the introduction of more recent studies and systematic reviews/meta-analyses (in this published literature) on this topic.

### Limitations

Our systematic review has certain limitations. One critical limitation is the lack of studies primarily investigating the association between statin use and the incidence of NODM. Consequently, these studies did not have the evaluation of NODM risk with statin use as a primary objective. Most of the published trials assessed NODM through a secondary analysis, and they were not powered to detect this association. Hence, there is a need to conduct specific studies that are powered to detect this association. A second limitation of our systematic review is that we cannot provide the exact number of participants included in our systematic review. This is due to the fact that most of the systematic reviews and meta-analyses included in our report contain data from the same or overlapping studies and RCTs. Another limitation of our systematic review is that some of the articles accessed may contain the same data, and bigger trials with large sample sizes (assessed in multiple published systematic reviews and meta-analyses) may bias the result, whereas a new systematic review would not be prone to this possibility. Lastly, the systematic reviews and meta-analyses included in this systematic review range from inception to 2019; hence, we may have missed certain reports published after the considered period. However, a systematic review can be updated after a few years depending on the number of studies published and the relevance of the data generated by the studies.

## Conclusions

Statin use is critical for the treatment and prevention of CVD.^37–40^ They are widely used, and their benefits have already been proven. It is not desirable to discourage their use in view of anticipating the risk of developing DM. Discontinuing statin therapy could be more harmful than the risk of developing DM. It is important to assess the risk of NODM and monitor the glycaemic parameters in subjects receiving statins. At the same time, the concerned authorities must develop risk assessment and mitigation strategies to ensure a safer use of statins. Different strategies, such as reducing the dose of individual statins, switching from one statin to another or, if needed, switching to non-statin hypolipidaemic drugs, may be considered.

From the findings of our systematic review, we can conclude that statin may cause NODM. Meanwhile, it is very important to understand that, at present, studies specifically addressing the relationship between statins and the incidence of NODM are lacking. Because statins are one of the most important agents used in dyslipidaemias and CVD, their use should not be discouraged in anticipation of the risk of NODM.

However, there is a need to encourage the monitoring of glycaemic parameters while the statin therapy is on-going and to conduct clinical studies specifically addressing the use of statins and the incidence of NODM as their primary objective.
